# Supplementary material for: Structural insights into the committed step of bacterial phospholipid biosynthesis
Source: Nat Commun. 2017 Nov 22;8:1691. doi: 10.1038/s41467-017-01821-9 (PMC5700162; doi:10.1038/s41467-017-01821-9)
Supplement: Supplementary file 1 — Supplementary Information [file 41467_2017_1821_MOESM1_ESM.pdf]

Supplementary Table 1. Sequences and primers.

Name	Sequence[a]
Aquifex aeolicus plsY (*aa_plsY*)[b]	GCGCTTTTCTTAGTTATTTTTGCTTATTTGTTGGGGAGCATCACTTTCGGCGAAGTTAT<br>CGCCAAACTTAAGGGCGTGGATTTGCGTAACGTGGGTTCAGGGAATGTTGGAGCTAC<br>TAATGTAACCCGTGCACTTGGCAAGAAATACGGGGTTTTGGTGTTTTTTCTTGACTTTT<br>TAAAAGGCTTCATTCCCGCACTGATTGCGGTGAAATCATTTGGGATCGACTCGTGGGT<br>TTTGACCTTCACTGGCCTTGCGAGTGTTCTGGGTCACATGTATCCAGTTTTCTTTGGGT<br>TTAAAGGAGGAAAAGGCGTTGCCACCGCATTAGGTGTTGTCTTCGCAGTGAGTCCTTC<br>AGTTGCTCTGTTCTCGTTCTTGGTCTGGTTGGGAATTTTCCTGTGGAAACGCTATGTGA<br>GTCTTGCATCTATCACCGCTACCATCTCCGCCTTTTTGTTTCTTTTCGTGGCTGGATACC<br>CAGTCAATGTGTTGTTTATGGCTATTGTTATTGGAGCTTTAATCATCTACCGTCATCGT<br>GAAAATATCAATCGTTTACTTACAGGACGTGAACATCGCTTT
*aa_plsY*_F1[c]	GGATCCGCGCTTTTCTTAGTTATTTTTGCTTATTTGTTGGGGAGCATCACTTTCGGCGA
*aa_plsY*_R1[c]	CACGTTACGCAAATCCACGCCCTTAAGTTTGGCGATAACTTCGCCGAAAGTGATGCTCC
*aa_plsY*_F2	CGTGGATTTGCGTAACGTGGGTTCAGGGAATGTTGGAGCTACTAATGTAACCCGTGCAC
*aa_plsY*_R2	AAGTCAAGAAAAAACACCAAAACCCCGTATTTCTTGCCAAGTGCACGGGTTACATTAGT
*aa_plsY*_F3	TGGTGTTTTTTCTTGACTTTTTAAAAGGCTTCATTCCCGCACTGATTGCGGTGAAATCA
*aa_plsY*_R3	CAAGGCCAGTGAAGGTCAAAACCCACGAGTCGATCCCAAATGATTTCACCGCAATCAGT
*aa_plsY*_F4	TTGACCTTCACTGGCCTTGCGAGTGTTCTGGGTCACATGTATCCAGTTTTCTTTGGGTT
*aa_plsY*_R4	GACAACACCTAATGCGGTGGCAACGCCTTTTCCTCCTTTAAACCCAAAGAAAACTGGAT
*aa_plsY*_F5	CACCGCATTAGGTGTTGTCTTCGCAGTGAGTCCTTCAGTTGCTCTGTTCTCGTTCTTGG
*aa_plsY*_R5	AGACTCACATAGCGTTTCCACAGGAAAATTCCCAACCAGACCAAGAACGAGAACAGAGC
*aa_plsY*_F6	GGAAACGCTATGTGAGTCTTGCATCTATCACCGCTACCATCTCCGCCTTTTTGTTTCTT
*aa_plsY*_R6	TAGCCATAAACAACACATTGACTGGGTATCCAGCCACGAAAAGAAACAAAAAGGCGGAG
*aa_plsY*_F7	AATGTGTTGTTTATGGCTATTGTTATTGGAGCTTTAATCATCTACCGTCATCGTGAAAA
*aa_plsY*_R7	GGTACCAAAGCGATGTTCACGTCCTGTAAGTAAACGATTGATATTTTCACGATGACGGTAGA
Streptococcus pneumonia plsY (*sp_plsY*)[b]	ATCACTATTGTATTATTGATCTTAGCTTACCTGTTGGGGTCAATTCCGAGCGGCCTTTG<br>GATCGGTCAAGTGTTTTTCCAAATTAATTTGCGTGAGCATGGCTCAGGTAATACGGGG<br>ACCACCAATACATTTCGCATCTTGGGTAAAAAGGCCGGGATGGCTACCTTTGTCATCG<br>ATTTCTTTAAGGGTACGCTGGCCACGTTGCTGCCCATTATCTTTCACCTTCAAGGAGTC<br>AGCCCCCTTATTTTCGGCTTATTGGCAGTTATTGGCCACACTTTCCCCATTTTCGCCGG<br>ATTTAAGGGTGGCAAAGCCGTTGCAACTAGCGCGGGTGTCATTTTCGGATTTGCTCCC<br>ATTTTCTGCCTTTACTTGGCTATTATCTTCTTTGGAGCCTTATACTTGGGATCAATGAT<br>CTCGTTGTCTTCGGTCACGGCAAGTATCGCAGCGGTAATTGGAGTGTTGTTGTTCCCTT<br>TATTCGGCTTCATCCTGTCAAATTATGACTCATTATTTATTGCTATCATCCTGGCGTTA<br>GCATCCCTGATCATCATTCGCCATAAGGATAACATCGCTCGCATTAAAAACAAAACA<br>GAAAACCTTGTCCCCTGGGGTCTGAATTTAACACACCAAGATCCTAAGAAG
*sp_plsY*_F1[c]	GGATCCATCACTATTGTATTATTGATCTTAGCTTACCTGTTGGGGTCAATTCCGAGCGG
*sp_plsY*_R1	ACGCAAATTAATTTGGAAAAACACTTGACCGATCCAAAGGCCGCTCGGAATTGACCCCA
*sp_plsY*_F2	TTTCCAAATTAATTTGCGTGAGCATGGCTCAGGTAATACGGGGACCACCAATACATTTC
*sp_plsY*_R2	ATGACAAAGGTAGCCATCCCGGCCTTTTTACCCAAGATGCGAAATGTATTGGTGGTCCC
*sp_plsY*_F3	GGATGGCTACCTTTGTCATCGATTTCTTTAAGGGTACGCTGGCCACGTTGCTGCCCATT
*sp_plsY*_R3	AGCCGAAAATAAGGGGGCTGACTCCTTGAAGGTGAAAGATAATGGGCAGCAACGTGGCC
*sp_plsY*_F4	AGCCCCCTTATTTTCGGCTTATTGGCAGTTATTGGCCACACTTTCCCCATTTTCGCCGG
*sp_plsY*_R4	GACACCCGCGCTAGTTGCAACGGCTTTGCCACCCTTAAATCCGGCGAAAATGGGGAAAG
*sp_plsY*_F5	TGCAACTAGCGCGGGTGTCATTTTCGGATTTGCTCCCATTTTCTGCCTTTACTTGGCTA
*sp_plsY*_R5	GAGATCATTGATCCCAAGTATAAGGCTCCAAAGAAGATAATAGCCAAGTAAAGGCAGAA
*sp_plsY*_F6	ACTTGGGATCAATGATCTCGTTGTCTTCGGTCACGGCAAGTATCGCAGCGGTAATTGGA
*sp_plsY*_R6	AATTTGACAGGATGAAGCCGAATAAAGGGAACAACAACACTCCAATTACCGCTGCGATA
*sp_plsY*_F7	GGCTTCATCCTGTCAAATTATGACTCATTATTTATTGCTATCATCCTGGCGTTAGCATC
*sp_plsY*_R7	AATGCGAGCGATGTTATCCTTATGGCGAATGATGATCAGGGATGCTAACGCCAGGATGA
*sp_plsY*_F8	GGATAACATCGCTCGCATTAAAAACAAAACAGAAAACCTTGTCCCCTGGGGTCTGAATT
*sp_plsY*_R8	GGTACCCTTCTTAGGATCTTGGTGTGTTAAATTCAGACCCCAGGGGAC

Supplementary Table 1. Sequences and primers (continued).

Name	Sequence[a]
Thermotoga maritime plsY (*tm_plsY*)[b]	ATTTTAGGCTATTTCATTGGGTCTATCCCTTTCTCTTATTTGATCCCTAAATGGTTAAA<br>GGGAATCGACGTTCGTAAGGTGGGAAGTGGCAATGTAGGGGCCACCAATGCCATTCG<br>CACTACGGGCCCAGCAGTCGGGGGTATCTGCTTGCTGCTTGACGCCTTGAAAGGGTTC<br>TTTCCCGTGTTTATTACTATCACTTTCAGCGGAGACTCTAAAATTGTCTCGTTAACAGC<br>TATCGCAACTGTTTTGGGGCACGACTTTCCAATCTTCATGAAGTTCAAGGGAGGTAAA<br>GGCGTAGCGAGCACCCTGGGTATCATTTTCTGTCTGTCGTGGCCCACAGGGTTAGTCT<br>TCACCTTGACATGGCTGGTTATCGTCATGTTAACAAAATACGCCTCTCTGGGAAGTTT<br>AGTGGCCCTGTACGTTAGTGCATTATTAGGTTACCTTCTGAAGGGATACGACACAGGG<br>ATGCTTTTTTTGATCTTGGCAGTCCTGTCTACTTTGCGCCATTCCGAGAACATTCAACG<br>TTTATTAAATGGAACGGAACGTAAGGTG
*tm_plsY*_F1[c]	GGATCCATTTTAGGCTATTTCATTGGGTCTATCCCTTTCTCTTATTTGATCCCTAAATG
*tm_plsY*_R1	ATTGCCACTTCCCACCTTACGAACGTCGATTCCCTTTAACCATTTAGGGATCAAATAAG
*tm_plsY*_F2	TAAGGTGGGAAGTGGCAATGTAGGGGCCACCAATGCCATTCGCACTACGGGCCCAGCAG
*tm_plsY*_R2	AACCCTTTCAAGGCGTCAAGCAGCAAGCAGATACCCCCGACTGCTGGGCCCGTAGTGCG
*tm_plsY*_F3	TTGACGCCTTGAAAGGGTTCTTTCCCGTGTTTATTACTATCACTTTCAGCGGAGACTCT
*tm_plsY*_R3	GCCCCAAAACAGTTGCGATAGCTGTTAACGAGACAATTTTAGAGTCTCCGCTGAAAGTG
*tm_plsY*_F4	ATCGCAACTGTTTTGGGGCACGACTTTCCAATCTTCATGAAGTTCAAGGGAGGTAAAGG
*tm_plsY*_R4	CCACGACAGACAGAAAATGATACCCAGGGTGCTCGCTACGCCTTTACCTCCCTTGAACT
*tm_plsY*_F5	CATTTTCTGTCTGTCGTGGCCCACAGGGTTAGTCTTCACCTTGACATGGCTGGTTATCG
*tm_plsY*_R5	GCCACTAAACTTCCCAGAGAGGCGTATTTTGTTAACATGACGATAACCAGCCATGTCAA
*tm_plsY*_F6	CTCTGGGAAGTTTAGTGGCCCTGTACGTTAGTGCATTATTAGGTTACCTTCTGAAGGGA
*tm_plsY*_R6	ACAGGACTGCCAAGATCAAAAAAAGCATCCCTGTGTCGTATCCCTTCAGAAGGTAACCT
*tm_plsY*_F7	TTGATCTTGGCAGTCCTGTCTACTTTGCGCCATTCCGAGAACATTCAACGTTTATTAAA
*tm_plsY*_R7	GGTACCCACCTTACGTTCCGTTCCATTTAATAAACGTTGAATGT
Thermus thermophiles (*tt_plsY*)[b]	ACGGCGGCTGTTTGGACACTTCTGTTGGCCTACCTTTTTGGGAGCGTACCCGCTGGCG<br>TACTGGTCGCTCGTACTTATGGAGTTGATCTGCGTAAGGTAGGTTCTGGAAATATTGG<br>AGCGACTAACGTCTTACGCGCGTTGGGATGGGGACCCGCCCTTGTAGTGGCATTTTTC<br>GACGTGTTTAAAGGGGGAATTGCGGTATTGGTGGCCCGCGCATTTGGGTTATCTGACT<br>GGATGTTGGGTGGGGTCGCTTTAATGGCTGTGTTAGGCCATAATTATAGTGTCTTTTTG<br>CGCTTTCGCGGCGGGAAGGGTGTTGCGACGTCCTTTGGCACTCTTTTATTTCTTGATCC<br>AGTGCTGGCACTGTGGACGTTTCCGATTGGCTTATCGGTAATTCTTCTGACCCGCTATG<br>TTTCAGCCGGCTCGATGACTGGCGGTGTTGCCGCGTTCGTTTTGTCACTTGCCCTGGGA<br>CGCCCTCTGTGGGAGGTAGCAACGGTATTCTTGATGGCTTTACTTATTTCTGGACGC<br>ATCGTGAAAATCTTAAACGCCTTCGTGAAGGGACAGAGCGTCGTCTTGGGGAACGCG<br>TGGAAGCCCGC
*tt_plsY*_F1[c]	GGATCCACGGCGGCTGTTTGGACACTTCTGTTGGCCTACCTTTTTGGGAGCGTACCCGC
*tt_plsY*_R1	ACGCAGATCAACTCCATAAGTACGAGCGACCAGTACGCCAGCGGGTACGCTCCCAAAAA
*tt_plsY*_F2	TTATGGAGTTGATCTGCGTAAGGTAGGTTCTGGAAATATTGGAGCGACTAACGTCTTAC
*tt_plsY*_R2	AAAAATGCCACTACAAGGGCGGGTCCCCATCCCAACGCGCGTAAGACGTTAGTCGCTCC
*tt_plsY*_F3	CCCTTGTAGTGGCATTTTTCGACGTGTTTAAAGGGGGAATTGCGGTATTGGTGGCCCGC
*tt_plsY*_R3	AAGCGACCCCACCCAACATCCAGTCAGATAACCCAAATGCGCGGGCCACCAATACCGCA
*tt_plsY*_F4	ATGTTGGGTGGGGTCGCTTTAATGGCTGTGTTAGGCCATAATTATAGTGTCTTTTTGCG
*tt_plsY*_R4	AGTGCCAAAGGACGTCGCAACACCCTTCCCGCCGCGAAAGCGCAAAAAGACACTATAAT
*tt_plsY*_F5	TGCGACGTCCTTTGGCACTCTTTTATTTCTTGATCCAGTGCTGGCACTGTGGACGTTTC
*tt_plsY*_R5	GAAACATAGCGGGTCAGAAGAATTACCGATAAGCCAATCGGAAACGTCCACAGTGCCAG
*tt_plsY*_F6	TTCTGACCCGCTATGTTTCAGCCGGCTCGATGACTGGCGGTGTTGCCGCGTTCGTTTTG
*tt_plsY*_R6	CCGTTGCTACCTCCCACAGAGGGCGTCCCAGGGCAAGTGACAAAACGAACGCGGCAACA
*tt_plsY*_F7	CTGTGGGAGGTAGCAACGGTATTCTTGATGGCTTTACTTATTTCTGGACGCATCGTGA
*tt_plsY*_R7	GGTACCGCGGGCTTCCACGCGTTCCCCAAGACGACGCTCTGTCCCTTCACGAAGGCG<br>TTTAAGATTTTCACGATGCGTCCAGAAAA

Supplementary Table 1. Sequences and primers (continued).

Name	Sequence[a]
*ec_plsY*_F[d]	AGGCCTCTGCAGTCGACGGGCCCG*GGATCC*AGTGCAATCGCGCCTGG
*ec_plsY*_R	CGGCCAGTGAATTCGAGCTC*GGTACC*CTCGGGGTCCTTTTCGCG
S35A_F[e]	GGATTTGCGTAACGTGGGTGCAGGGAATGTTGGAGCTAC
S35A_R	GTAGCTCCAACATTCCCTGCACCCACGTTACGCAAATCC
S35C_F	GATTTGCGTAACGTGGGTTGCGGGAATGTTGGAGCTAC
S35C_R	GTAGCTCCAACATTCCCGCAACCCACGTTACGCAAATC
S35T_F	GATTTGCGTAACGTGGGTACAGGGAATGTTGGAGCTAC
S35T_R	GTAGCTCCAACATTCCCTGTACCCACGTTACGCAAATC
N37A_F	GCGTAACGTGGGTTCAGGGGCTGTTGGAGCTACTAATGTAAC
N37A_R	GTTACATTAGTAGCTCCAACAGCCCCTGAACCCACGTTACGC
N37D_F	GCGTAACGTGGGTTCAGGGGATGTTGGAGCTACTAATGTAAC
N37D_R	GTTACATTAGTAGCTCCAACATCCCCTGAACCCACGTTACGC
N37H_F	GCGTAACGTGGGTTCAGGGCATGTTGGAGCTACTAATGTAAC
N37H_R	GTTACATTAGTAGCTCCAACATGCCCTGAACCCACGTTACGC
N37Q_F	CGTAACGTGGGTTCAGGGCAGGTTGGAGCTACTAATGTAAC
N37Q_R	GTTACATTAGTAGCTCCAACCTGCCCTGAACCCACGTTACG
N37S_F	GCGTAACGTGGGTTCAGGGAGTGTTGGAGCTACTAATGTAAC
N37S_R	GTTACATTAGTAGCTCCAACACTCCCTGAACCCACGTTACGC
T41A_F	GTTCAGGGAATGTTGGAGCTGCTAATGTAACCCGTGCACTTG
T41A_R	CAAGTGCACGGGTTACATTAGCAGCTCCAACATTCCCTGAAC
T41S_F	TTCAGGGAATGTTGGAGCTTCTAATGTAACCCGTGCACTTG
T41S_R	CAAGTGCACGGGTTACATTAGAAGCTCCAACATTCCCTGAA
R45A_F	GGAGCTACTAATGTAACCGCTGCACTTGGCAAGAAATAC
R45A_R	GTATTTCTTGCCAAGTGCAGCGGTTACATTAGTAGCTCC
R45K_F	GGAGCTACTAATGTAACCAAGGCACTTGGCAAGAAATAC
R45K_R	GTATTTCTTGCCAAGTGCCTTGGTTACATTAGTAGCTCC
H92A_F	CTTGCGAGTGTTCTGGGTGCCATGTATCCAGTTTTCTTTGG
H92A_R	CCAAAGAAAACTGGATACATGGCACCCAGAACACTCGCAAG
H92D_F	GGCCTTGCGAGTGTTCTGGGTGACATGTATCCAGTTTTCTTTGGG
H92D_R	CCCAAAGAAAACTGGATACATGTCACCCAGAACACTCGCAAGGCC
H92E_F	GGCCTTGCGAGTGTTCTGGGTGAAATGTATCCAGTTTTCTTTGGG
H92E_R	CCCAAAGAAAACTGGATACATTTCACCCAGAACACTCGCAAGGCC
H92K_F	CCTTGCGAGTGTTCTGGGTAAAATGTATCCAGTTTTCTTTG
H92K_R	CAAAGAAAACTGGATACATTTTACCCAGAACACTCGCAAGG
H92L_F	CTTGCGAGTGTTCTGGGTCTTATGTATCCAGTTTTCTTTG
H92L_R	CAAAGAAAACTGGATACATAAGACCCAGAACACTCGCAAG
H92N_F	CCTTGCGAGTGTTCTGGGTAATATGTATCCAGTTTTCTTTG
H92N_R	CAAAGAAAACTGGATACATATTACCCAGAACACTCGCAAGG
H92Q_F	CCTTGCGAGTGTTCTGGGTCAGATGTATCCAGTTTTCTTTG
H92Q_R	CAAAGAAAACTGGATACATCTGACCCAGAACACTCGCAAGG
G102A_F	GTATCCAGTTTTCTTTGGGTTTAAAGCAGGAAAAGGCGTTGCCACCGC
G102A_R	GCGGTGGCAACGCCTTTTCCTGCTTTAAACCCAAAGAAAACTGGATAC
G103A_F	GTATCCAGTTTTCTTTGGGTTTAAAGGAGCAAAAGGCGTTGCCACCGCATTAG
G103A_R	CTAATGCGGTGGCAACGCCTTTTGCTCCTTTAAACCCAAAGAAAACTGGATAC
K104A_F	CTTTGGGTTTAAAGGAGGAGCAGGCGTTGCCACCGCATTAG
K104A_R	CTAATGCGGTGGCAACGCCTGCTCCTCCTTTAAACCCAAAG
K104R_F	CTTTGGGTTTAAAGGAGGACGTGGCGTTGCCACCGCATTAG
K104R_R	CTAATGCGGTGGCAACGCCACGTCCTCCTTTAAACCCAAAG
G105A_F	CTTTGGGTTTAAAGGAGGAAAAGCCGTTGCCACCGCATTAGG
G105A_R	CCTAATGCGGTGGCAACGGCTTTTCCTCCTTTAAACCCAAAG

Supplementary Table 1. Sequences and primers (continued).

Name	Sequence[a]
G105P_F	CTTTGGGTTTAAAGGAGGAAAACCAGTTGCCACCGCATTAGGTG
G105P_R	CACCTAATGCGGTGGCAACTGGTTTTCCTCCTTTAAACCCAAAG
V106G_F	GTTTAAAGGAGGAAAAGGCGGTGCCACCGCATTAGGTGTTG
V106G_R	CAACACCTAATGCGGTGGCACCGCCTTTTCCTCCTTTAAAC
V106P_F	GTTTAAAGGAGGAAAAGGCCCAGCCACCGCATTAGGTG
V106P_R	CACCTAATGCGGTGGCTGGGCCTTTTCCTCCTTTAAAC
A107P_F	GTTTAAAGGAGGAAAAGGCGTTCCCACCGCATTAGGTGTTGTCTTC
A107P_R	GAAGACAACACCTAATGCGGTGGGAACGCCTTTTCCTCCTTTAAAC
S142A_F	GAAACGCTATGTGAGTCTTGCAGCTATCACCGCTACCATCTCCGC
S142A_R	GCGGAGATGGTAGCGGTGATAGCTGCAAGACTCACATAGCGTTTC
H177A_F	GAGCTTTAATCATCTACCGTGCTCGTGAAAATATCAATCGTTTAC
H177A_R	GTAAACGATTGATATTTTCACGAGCACGGTAGATGATTAAAGCTC
H177D_F	GAGCTTTAATCATCTACCGTGATCGTGAAAATATCAATC
H177D_R	GATTGATATTTTCACGATCACGGTAGATGATTAAAGCTC
H177E_F	GAGCTTTAATCATCTACCGTGAACGTGAAAATATCAATC
H177E_R	GATTGATATTTTCACGTTCACGGTAGATGATTAAAGCTC
H177F_F	GGAGCTTTAATCATCTACCGTTTCCGTGAAAATATCAATCGTTTAC
H177F_R	GTAAACGATTGATATTTTCACGGAAACGGTAGATGATTAAAGCTCC
H177I_F	GGAGCTTTAATCATCTACCGTATCCGTGAAAATATCAATCGTTTAC
H177I_R	GTAAACGATTGATATTTTCACGGATACGGTAGATGATTAAAGCTCC
H177K_F	GAGCTTTAATCATCTACCGTAAACGTGAAAATATCAATCG
H177K_R	CGATTGATATTTTCACGTTTACGGTAGATGATTAAAGCTC
H177L_F	GGAGCTTTAATCATCTACCGTCTGCGTGAAAATATCAATCGTTTAC
H177L_R	GTAAACGATTGATATTTTCACGCAGACGGTAGATGATTAAAGCTCC
H177M_F	GGAGCTTTAATCATCTACCGTATGCGTGAAAATATCAATCGTTTAC
H177M_R	GTAAACGATTGATATTTTCACGCATACGGTAGATGATTAAAGCTCC
H177N_F	GCTTTAATCATCTACCGTAATCGTGAAAATATCAATCG
H177N_R	CGATTGATATTTTCACGATTACGGTAGATGATTAAAGC
H177Q_F	GGAGCTTTAATCATCTACCGTCAACGTGAAAATATCAATCGTTTAC
H177Q_R	GTAAACGATTGATATTTTCACGTTGACGGTAGATGATTAAAGCTCC
H177Y_F	GGAGCTTTAATCATCTACCGTTACCGTGAAAATATCAATCGTTTAC
H177Y_R	GTAAACGATTGATATTTTCACGGTAACGGTAGATGATTAAAGCTCC
N180A_F	CTACCGTCATCGTGAAGCTATCAATCGTTTACTTACAG
N180A_R	CTGTAAGTAAACGATTGATAGCTTCACGATGACGGTAG
N180D_F	CTACCGTCATCGTGAAGATATCAATCGTTTACTTACAG
N180D_R	CTGTAAGTAAACGATTGATATCTTCACGATGACGGTAG
N180H_F	CATCTACCGTCATCGTGAACATATCAATCGTTTACTTAC
N180H_R	GTAAGTAAACGATTGATATGTTCACGATGACGGTAGATG
N180Q_F	CATCTACCGTCATCGTGAACAAATCAATCGTTTACTTACAG
N180Q_R	CTGTAAGTAAACGATTGATTTGTTCACGATGACGGTAGATG
R183A_F	CCGTCATCGTGAAAATATCAATGCTTTACTTACAGGACGTGAACATCG
R183A_R	CGATGTTCACGTCCTGTAAGTAAAGCATTGATATTTTCACGATGACGG
R183E_F	CCGTCATCGTGAAAATATCAATGAATTACTTACAGGACGTGAACATCG
R183E_R	CGATGTTCACGTCCTGTAAGTAATTCATTGATATTTTCACGATGACGG
R183K_F	CCGTCATCGTGAAAATATCAATAAGTTACTTACAGGACGTGAACATCG
R183K_R	CGATGTTCACGTCCTGTAAGTAACTTATTGATATTTTCACGATGACGG
E189A_F	GTTTACTTACAGGACGTGCACATCGCTTTGGTACCCTG
E189A_R	CAGGGTACCAAAGCGATGTGCACGTCCTGTAAGTAAAC
E189D_F	GTTTACTTACAGGACGTGACCATCGCTTTGGTACCCTGG
E189D_R	CCAGGGTACCAAAGCGATGGTCACGTCCTGTAAGTAAAC
E189Q_F	CGTTTACTTACAGGACGTCAACATCGCTTTGGTACCCTG
E189Q_R	CAGGGTACCAAAGCGATGTTGACGTCCTGTAAGTAAACG

Supplementary Table 1. Sequences and primers (continued).

Name	Sequence[a]
*deoD*_gF[f]	AAGTGCTGTTTCAGGGCCCGGGATCCGCTACCCCACACATTAATGCAG
*deoD*_gR	GTTAGCAGCCGGATCTCAGGTACCCTCTTTATCGCCCAGCAGAAC
*deoD*_vF[f]	TGAGATCCGGCTGCTAACAAAGCCCG
*deoD*_vR	CGGGCCCTGAAACAGCACTTCCAG
PBP_gF[f]	CTTTAAGAAGGAGATATACCATGGAAGCAAGCCTGACAGGTGCAGGTG
PBP_gR	GACGGGCCCTGAAACAGCACTTCCAGGTACAGCGGCTTACCGCTACTGTC
PBP_vF[f]	CTGGAAGTGCTGTTTCAGGGCCCGTCTG
PBP_vR	CATGGTATATCTCCTTCTTAAAG
PBP_A197C_F	GTGCAATTGGTTACGTTGAATATTGTTACGCTAAGCAGAACAACCTGGCG
PBP_A197C_R	CGCCAGGTTGTTCTGCTTAGCGTAACAATATTCAACGTAACCAATTGCAC

[a]All DNA sequences are from the 5’ end to the 3’ end. [b]Sequences of indicated genes are codon-optimized for over-expression in *E. coli* host cells. [c]Overlapping primers for PCR-synthesis of indicated genes. For all the primers in this table, ‘F’ primers refer to the forward direction and ‘R’ primers refer to the reverse direction, with respect to the orientation of transcription. [d]The *E. coli plsY* gene was PCR-amplified with the two primers using genomic DNA as the template. [e]Primers for mutagenesis of the *aa_plsY* gene. [f]Primers for molecular cloning of the indicated genes using the Gibson Assembly method. A letter ‘g’ indicates primers for specific genes, and a letter ‘v’ indicates primers for vectors.

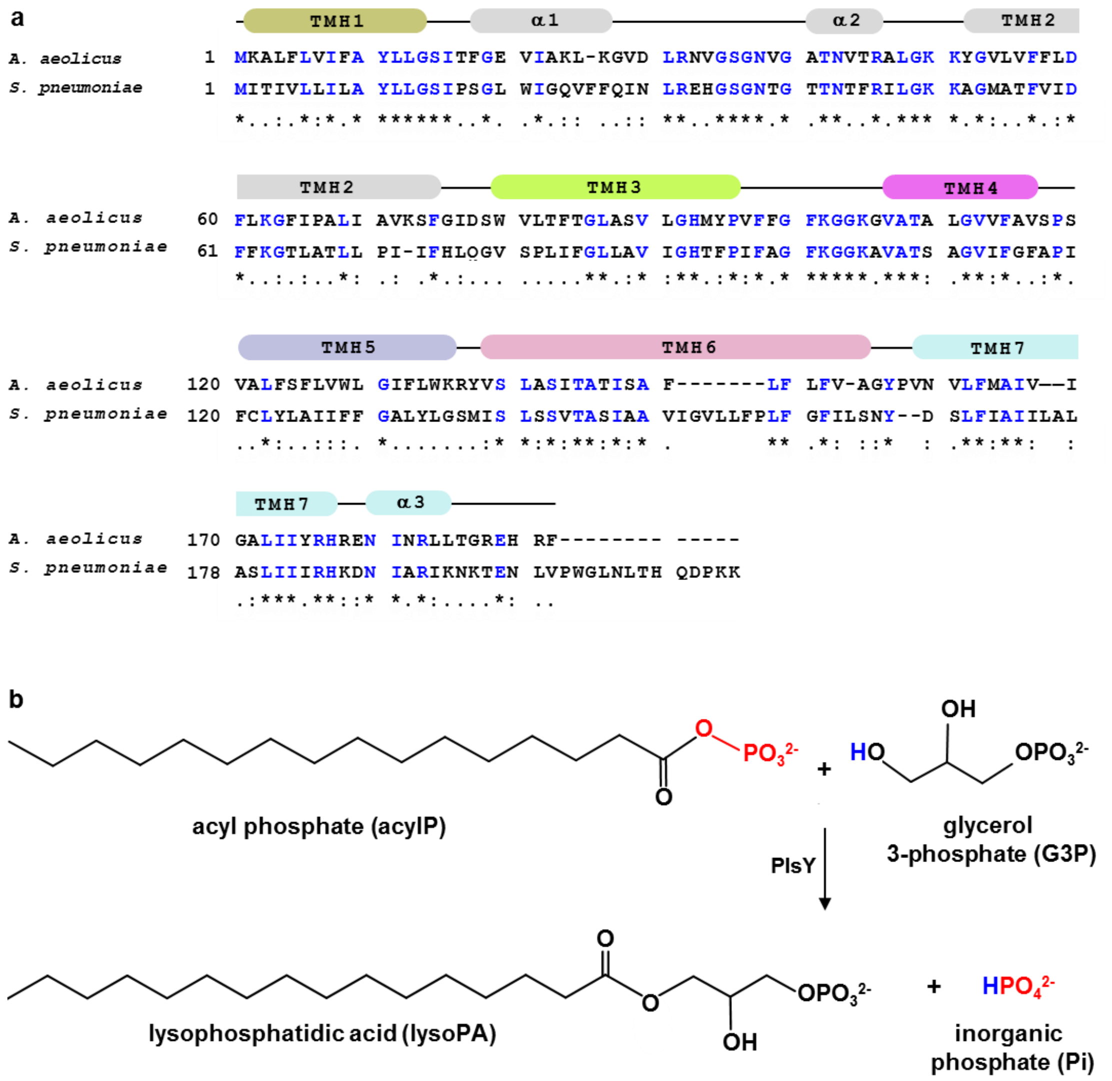

Supplementary Figure 1. Sequence alignment and reaction of PlsY. (**a**) Sequence alignment of *aa*PlsY and *sp*PlsY. The alignment was carried out using Needle[1]. The TMHs and three short α-helices were labeled above the sequences. (**b**) The reaction catalysed by PlsY.

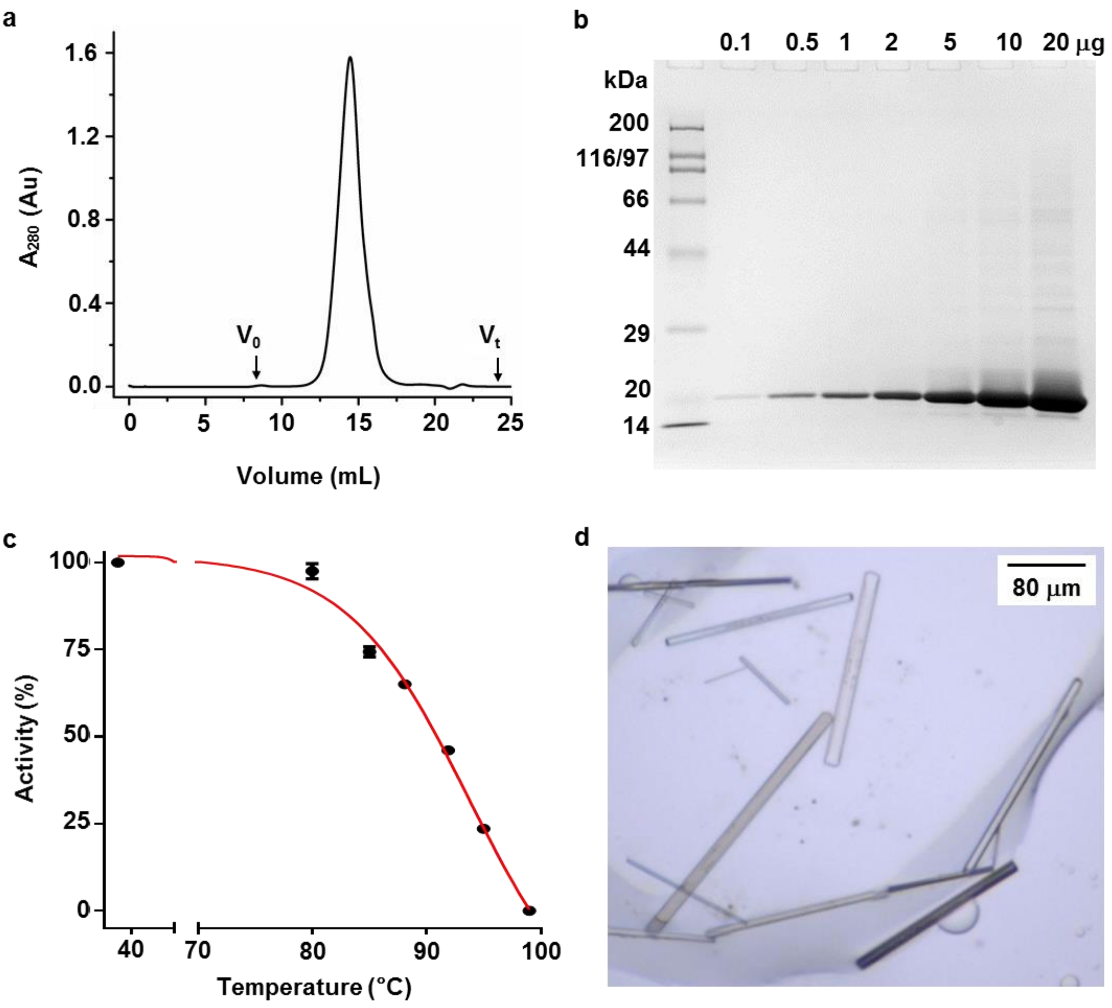

Supplementary Figure 2. Purification, stability assay and crystallization of *aa*PlsY. (**a**) Size exclusion chromatography of *aa*PlsY. V_o and V_t mark the void and total volume, respectively. (**b**) Purity test of *aa*PlsY by Coomassie-Blue stained SDS-PAGE. Loading amounts and molecular weight of markers are indicated appropriately. (**c**) Thermostability of *aa*PlsY. The protein solubilized in DDM was subjected to heat at indicated temperatures for 30 min prior to assay, except that the 99 ℃ sample was heated for 1 h. Data reported are the average ± standard deviation from triplicate measurements. (**d**) Crystals of *aa*PlsY in the LCP with 7.8 MAG as the host lipid.

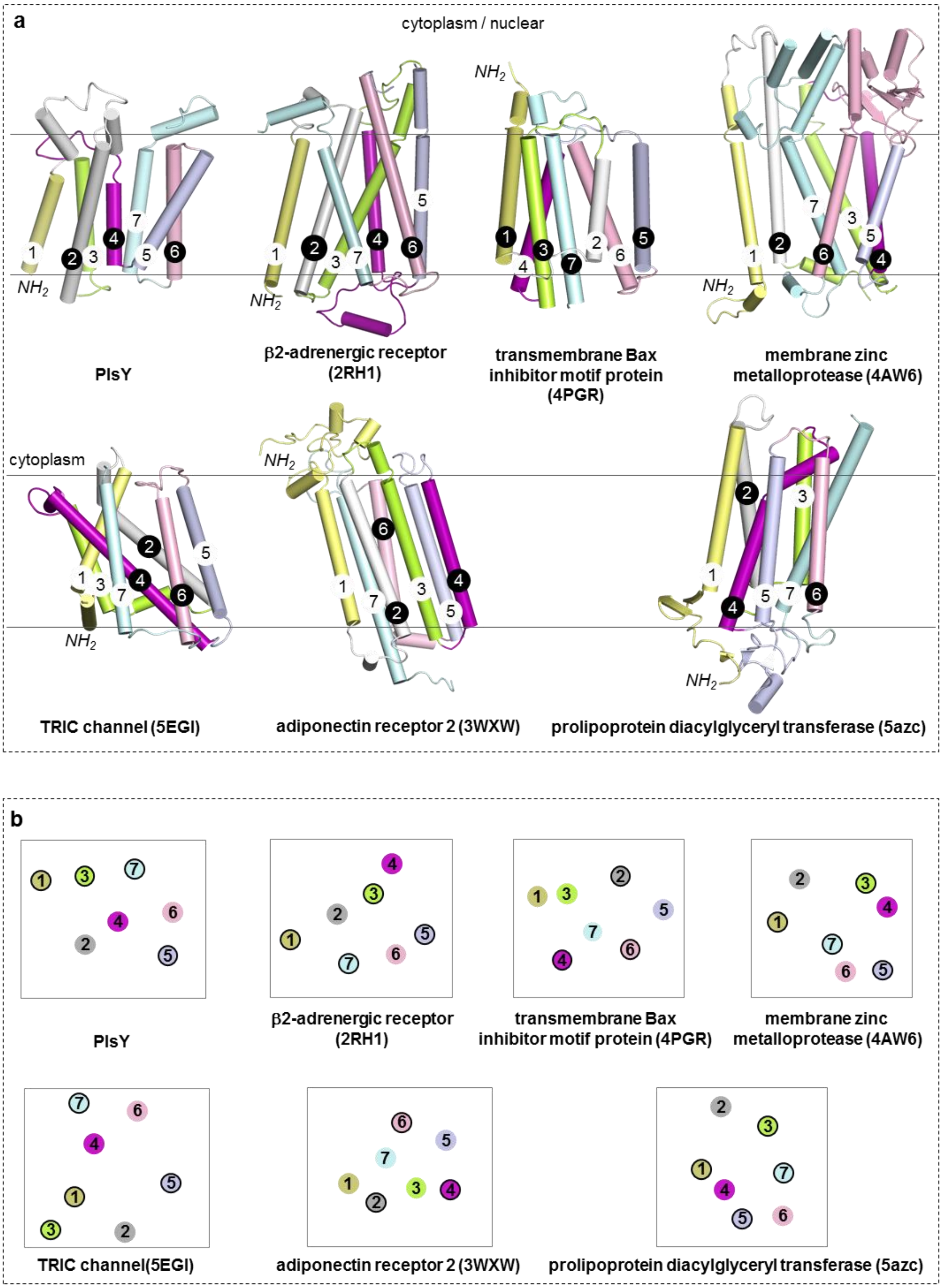

Supplementary Figure 3. Comparison of *aa*PlsY with known 7-TMHs structural folds. (**a**) View from the membrane. TMHs going towards cytoplasm (or nuclear for the membrane zinc metalloprotease) are numbered in white background. TMHs going away from cytoplasm are numbered in black background. (**b**) Schematic views from the cytoplasm. TMH numbers are shaded with the same colour as in **a**. A black boarder indicates TMHs that go towards the viewers (the cytoplasm). Names of the proteins are labeled below each structure, with the PDB entry IDs in brackets.

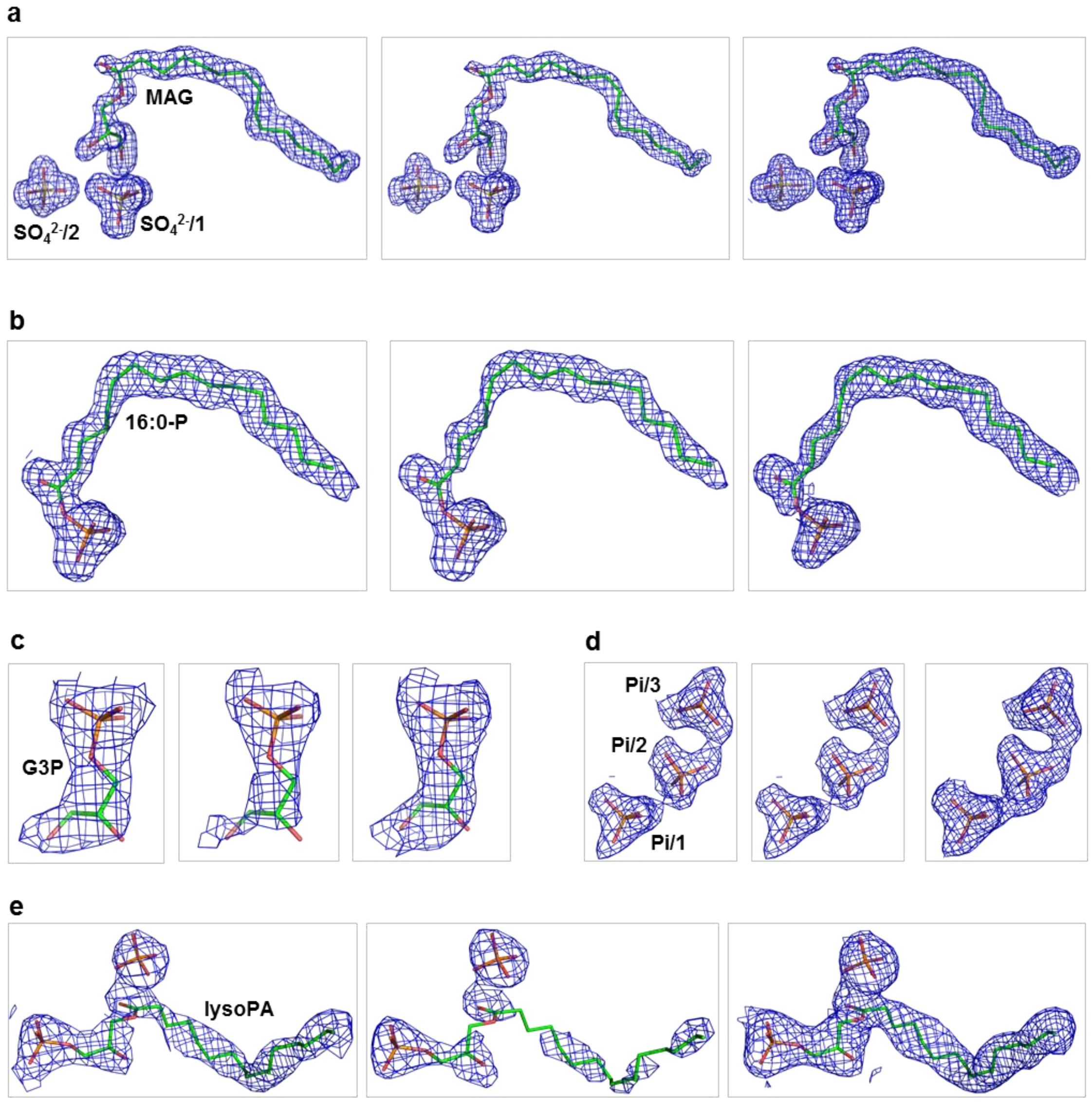

Supplementary Figure 4. Electron density maps of the active site ligands. Sigma-A weighted 2Fo-Fc simulated annealing (SA) omit map (left), Fo-Fc SA omit map (middle), and the composite omit map (right) contoured at 1.0, 3.0 and 1.0 σ levels, respectively, for MAG and SO_4^{2-} (**a**), 16:0-P (**b**), G3P (**c**), Pi, (**d**) and lysoPA (**e**).

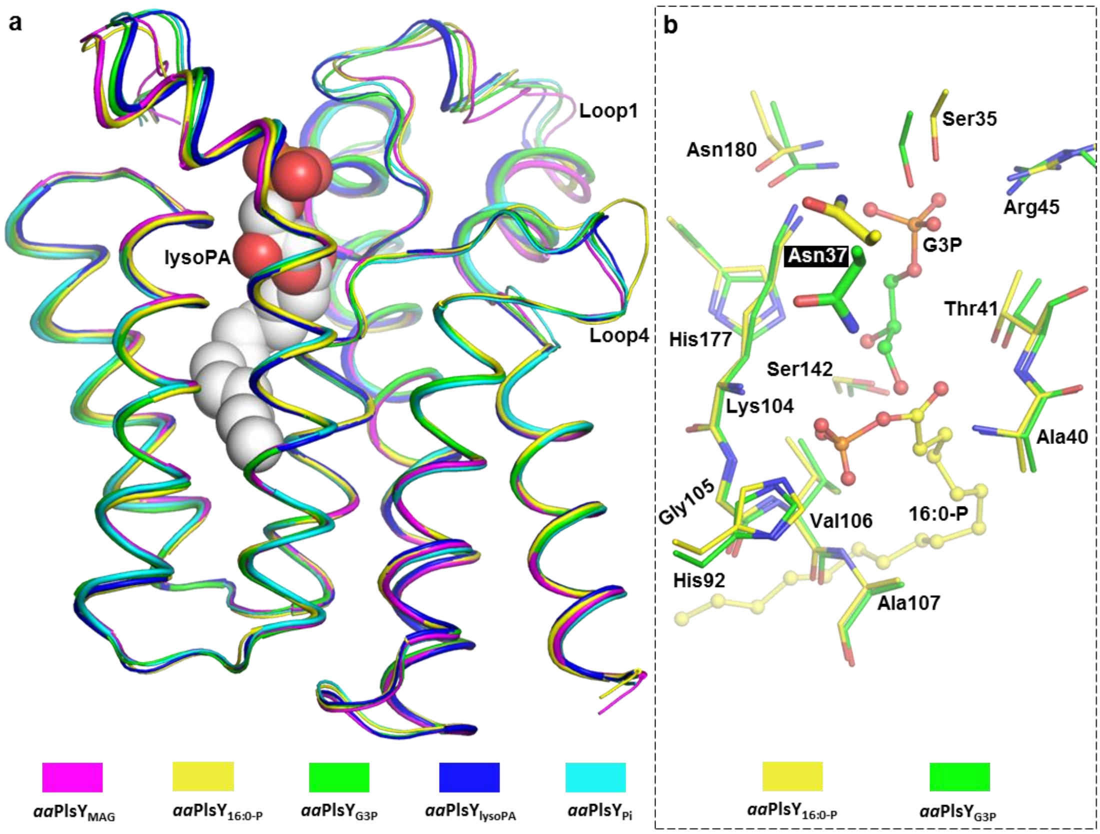

Supplementary Figure 5. Comparison of the *aa*PlsY structures. (**a**) The five superimposed structures. The structures are shown as ribbon representations with indicated colors. The root-mean-square deviation of substrate/product-bound structures from the *aa*PlsY$_{MAG}$ are 0.35 Å (*aa*PlsY$_{16:0\text{-}P}$), 0.37 Å (*aa*PlsY$_{G3P}$), 0.32 Å (*aa*PlsY$_{lysoPA}$) and 0.52 Å (*aa*PlsY$_{Pi}$). (**b**) The two superimposed active sites of *aa*PlsY$_{16:0\text{-}P}$ (yellow) and *aa*PlsY$_{G3P}$ (green). Asn37 interacts with neither G3P nor 16:0-P.

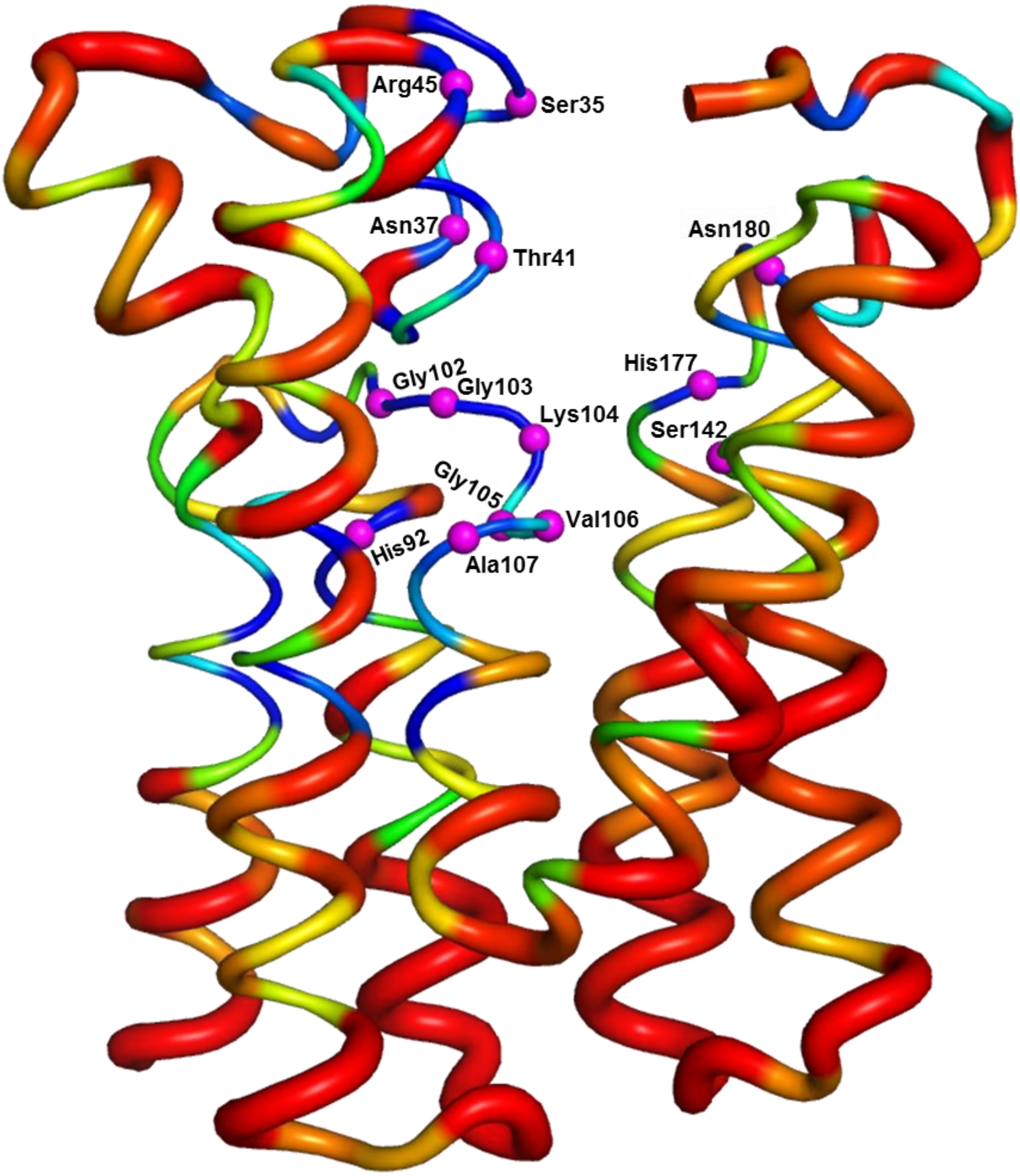

Supplementary Figure 6. Putty show of residue conservation in PlsY. The percentage frequency (%) of residues in 7,288 unique sequences from the InterPro 60.0 database[2] was subtracted from 100 and the values were mapped onto the *aa*PlsY$_{MAG}$ structure. Blue lines and red tube indicate high and low conservation, respectively. Functionally important residues are highlighted by showing the Cα atoms as magenta spheres.

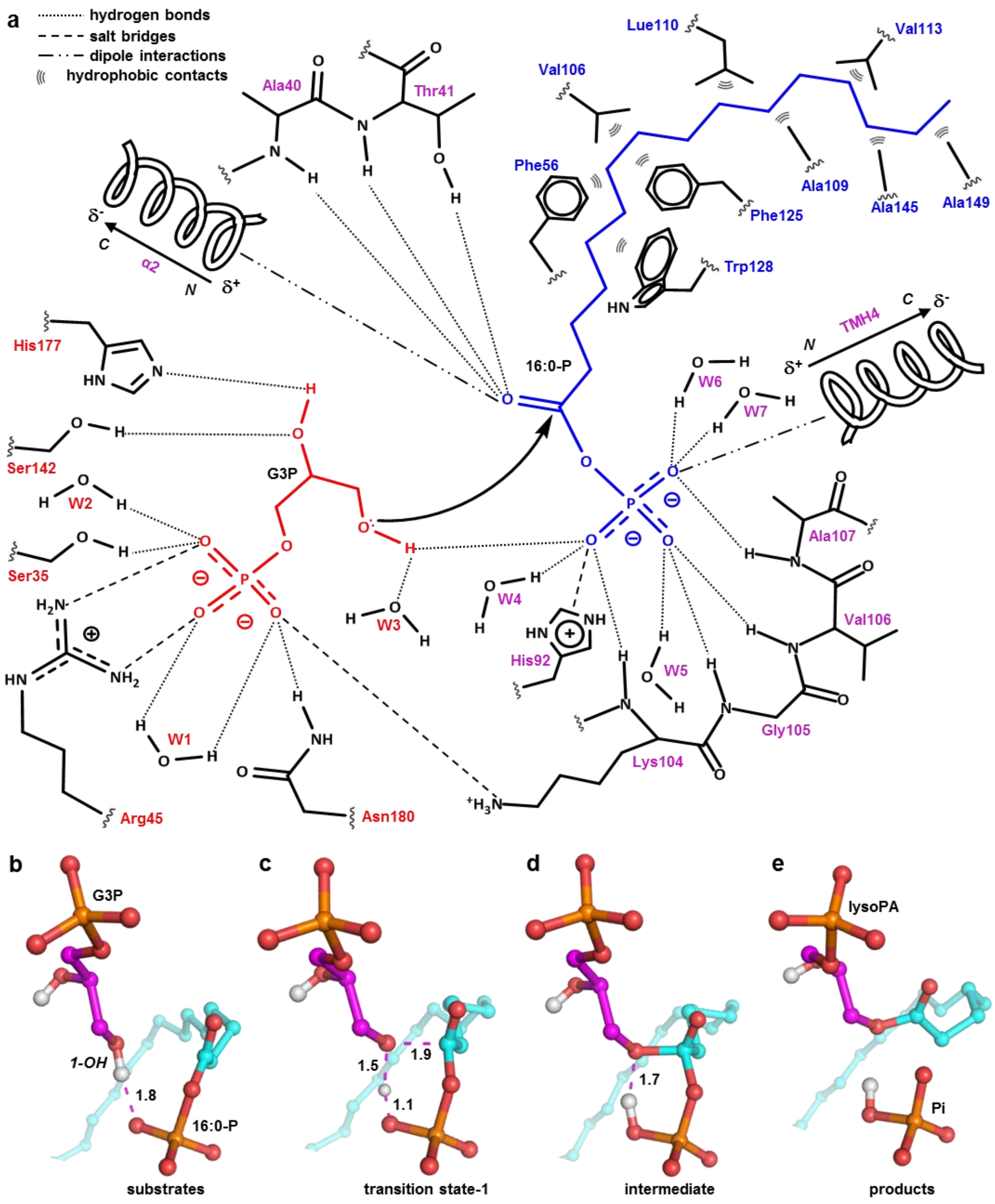

Supplementary Figure 7. Interactions between the substrates and *aa*PlsY, and quantum mechanics/molecular mechanics (QM/MM) optimized poses of the catalytic cycle. (a) Schematic drawing of the *aa*PlsY-substrate complex. (**b-e**) QM/MM optimized poses of the catalytic cycle. All steps except transition state-2 were obtained. Dashed lines indicate distances in Å. In the proposed mechanism, the 16:0-P phosphate forms an H-bond with G3P 1-OH (**b**), enhancing its nucleophilicity. The hydroxyl then attacks on the 16:0-P carbonyl, forming the **transition state-1** (**c**). Subsequently, the original O-H covalent bond ruptures as the proton shifts to the phosphate group, generating the tetrahedral oxyanion **intermediate** (**d**). In the following elimination steps, the tetrahedron collapses, recreating the carbonyl group, and finishing the catalytic cycle by ejecting the phosphate group (**e**).

Supplementary References

1 Li, W. *et al.* The EMBL-EBI bioinformatics web and programmatic tools framework. *Nucleic Acids Res* **43**, W580-584, (2015).

2 Finn, R. D. *et al.* InterPro in 2017—beyond protein family and domain annotations. *Nucleic Acids Res* **45**, D190-D199, (2017).
